# Draft Genome Sequence of *Streptomyces hygroscopicus* subsp. *hygroscopicus* NBRC 16556

**DOI:** 10.1128/genomeA.00139-16

**Published:** 2016-05-19

**Authors:** Hisayuki Komaki, Natsuko Ichikawa, Akio Oguchi, Moriyuki Hamada, Tomohiko Tamura, Ken-ichiro Suzuki, Nobuyuki Fujita

**Affiliations:** a Biological Resource Center, National Institute of Technology and Evaluation (NBRC), Chiba, Japan; b NBRC, Tokyo, Japan

## Abstract

Here, we report the draft genome sequence of strain NBRC 16556, deposited as *Streptomyces hygroscopicus* subsp. *hygroscopicus* into the NBRC culture collection. An average nucleotide identity analysis confirmed that the taxonomic identification is correct. The genome sequence will serve as a valuable reference for genome mining to search new secondary metabolites.

## GENOME ANNOUNCEMENT

*Streptomyces hygroscopicus* and related species are well known as promising sources of diverse secondary metabolites (1). Over 650 kinds of bioactive compounds were found from these members. *S. hygroscopicus* has three subspecies validly published so far: *S. hygroscopicus* subsp. *hygroscopicus*, *S. hygroscopicus* subsp. *glebosus*, and *S. hygroscopicus* subsp. *ossamyceticus*. Strain NBRC 16556 was originally isolated as strain A-5294 from soil in Tottori, Japan, by Kaken Chem. Co., found to produce vulgamycin (antibiotic WS-8096), and deposited as *S. hygroscopicus* subsp. *hygroscopicus* JCM 4885 into the NBRC culture collection in 2001. The scientific name was determined before 1983, when molecular-based identification methodologies were not general. Therefore, it was likely determined mainly based on morphological characteristics. Such a methodology is nowadays known to be less reliable than current genome-based identifications. To confirm that the taxonomic identification of this strain is correct, we conducted whole-genome shotgun sequencing.

Genomic DNA of strain NBRC 16556 was prepared from cultured mycelium using a Qiagen EZ1 tissue kit and an EZ1 Advanced instrument (Qiagen) and sequenced by a combined strategy of shotgun sequencing with GS FLX+ (Roche) and paired-end sequencing with MiSeq (Illumina). The reads (749 Mb sequences, 74-fold coverage) were assembled using Newbler version 2.6. Contigs obtained from the assembly were subsequently finished using GenoFinisher (2), which led to a final assembly of 113 scaffold sequences of >500 bp each. The total size of the assembly was 10,141,835 bp, with a G+C content of 72.0%.

We also sequenced the whole genome of a type strain (NBRC 13472) of *S. hygroscopicus* subsp. *hygroscopicus* and recently released the draft genome sequence under the accession number BBOX01000000. Then, we calculated the average nucleotide identity according to BLAST (ANIb) value between strains NBRC 16556 and NBRC 13472^T^ using their genome sequences. The value was 97.17%, which is above the threshold (95 to 96%) corresponding to DNA-DNA relatedness value of 70% recommended as the cutoff point for the assignment of bacterial strains to the same species (3, 4). Therefore, it is concluded that strain NBRC 16556 is definitely *S. hygroscopicus* subsp. *hygroscopicus*.

We publish here the draft genome sequence of *S. hygroscopicus* subsp. *hygroscopicus* NBRC 16556. The genome sequence will serve as a valuable reference for elucidating the diversity of the *S. hygroscopicus* group and for genome mining to search new secondary metabolites.

### Nucleotide sequence accession numbers.

The draft genome sequence of *S. hygroscopicus* subsp. *hygroscopicus* NBRC 16556 has been deposited in DDBJ/ENA/GenBank databases under the accession no. BBOU00000000. The version described in this paper is the first version, BBOU01000000.
